# CO-RADS score and its correlation with clinical and laboratory parameters in patients with COVID-19

**DOI:** 10.1186/s43168-022-00176-0

**Published:** 2023-01-09

**Authors:** Marwa Elsayed Elnaggar, Abeer Mohamed Rawy, Marwa Seif El-Melouk, Al-Shaimaa Mahmoud Al-Tabbakh, Hamasat Abdel-hafeez Abdel-Khalik, Eman Fathy Abdelkhalek, Rehab Elsayed Elsawy

**Affiliations:** 1 Chest Diseases, Faculty of Medicine, Benha University, Benha, Egypt; 2 Medical Microbiology and Immunology, Faculty of Medicine, Benha University, Benha, Egypt; 3 Clinical and Chemical Pathology, Faculty of Medicine, Benha University, Benha, Egypt; 4 Diagnostic and Interventional Radiology, Faculty of Medicine, Benha University, Benha, Egypt

**Keywords:** COVID-19, ALC, CT, CO-RADS

## Abstract

**Background:**

Polymerase chain reaction (PCR) based SARS-CoV-2 RNA detection and serological antibody tests give a proof of Coronavirus Disease 2019 (COVID-19) infection. Several variables can influence the consequences of these tests. Inflammatory markers among mild and severe patients of COVID-19 showed dissimilarity in inflammatory markers while computed tomography (CT) in patients infected with COVID-19 used to evaluate infection severity. The aim of this study is to investigate the application of the COVID-19 Reporting and Data System (CO-RADS) classification in COVID-19 patients and its relation to clinical and laboratory finding.

**Results:**

One hundred patients suspected to have COVID-19 infection were involved. Their age was 49.6 ± 14.7. Fever and cough were the frequent presenting symptoms. Patients with positive PCR were significantly associated with dyspnea and higher inflammatory markers. Lymphopenia had sensitivity of 63.6% and specificity of 91.7%. Combination of PCR and lymphopenia increased both sensitivity and specificity. CT findings in relation to PCR showed sensitivity of 90.5% and specificity of 25%. CO-RADS score showed positive correlation with age and inflammatory biomarkers and negative correlation with absolute lymphocyte count (ALC).

**Conclusions:**

CT finding was more prominent in older patients with COVID-19 and associated with higher inflammatory biomarkers and lower ALC which were correlated with CO-RADS score. Patients with positive PCR had more symptoms and inflammatory marker. Combination of PCR with either lymphopenia or CT finding had more sensitivity, specificity and accuracy in diagnosis

## Introduction

Close to the completion of 2019, a flare-up of pneumonia brought about by a novel human coronavirus (severe acute respiratory syndrome coronavirus 2 (SARS-CoV-2)) showed up in Wuhan, in the Hubei area in China. The novel emerging virus has spread quickly across China and all through the world [1]. At present, polymerase chain reaction (PCR) based SARS-CoV-2 RNA discovery from respiratory samples and serological antibody tests give immediate proof of Coronavirus Disease 2019 (COVID-19) infection [2].

Some variables can influence the consequences of PCR and serological antibody tests [3]. Additionally, the strength of the immune response is a typical component that influences both PCR and serological antibodies test by the capacities of virus clearance and creation of antibodies [4]. A hindered immune system is the overwhelming element of COVID-19 infection, as proven by an immediate upregulated inflammatory reaction leading to the following inflammatory storm [5].

Lymphopenia and elevated lactate dehydrogenase (LDH) were related to higher rate of intensive care unit (ICU) entry. Patients who were transmitted to the ICU had a lower nadir count of lymphocyte, monocyte, and hemoglobin, higher neutrophil (NEU) count and LDH levels contrasted with patients who did not need ICU stay [6].

A non-contrast high-resolution CT chest imaging assumes a crucial and fundamental part in the early disease identification, especially in patients with misleading negative RT-PCR results, as well as in directing and observing the course of disease [7].

Radiological evaluation of patients with SARS-Cov-2 infection particularly by chest computed tomography (CT) has a reported high sensitivity and enhances the clinical decision that is based on the degree of lung affection [8]. Moreover, the COVID-19 Reporting and Data System (CO-RADS) included information of clinical and labs finding that add to CT records. The level of suspicion ranged from very low to very high (CO-RADS categories 1–5), while classification 0 indicates negative infection and category 6 lays out RT-PCR-positive SARS-Cov-2 infection at time of assessment [9].

The purpose of this study is to investigate the application of the CO-RADS classification in COVID-19 patients and its relation to clinical and laboratory finding.

### Patients and methods

This prospective observational study was done in Benha University hospital on 100 patients presented with fever and/or respiratory symptom suggesting COVID-19 infection in the period between January and June 2021. The patients were assessed in the emergency department, outpatient clinic or admitted at chest department. The study was approved by Ethical Committee of Benha University, Faculty of Medicine (No. RC 5-6-2021). A written informed consent was obtained from all participants.

All patients were subjected to full medical history and clinical examination, nasopharyngeal swabs were taken by health care providers. Specimens were placed into viral transport medium (VTM) immediately after collection to preserve viral ribonucleic acid (RNA). Real-time polymerase chain reaction (RT-PCR) assay for SARS-CoV-2; RNA extraction was done using (QIAamp® viral RNA Mini Kit lot no 166029612 QIAGEN GmbH, Germany) then viral RNA detection by DTprime 4M1-DNAA technology, SN A5G206 Russia) following the manufacturer instruction of (The genesig® Real-Time PCR Coronavirus COVID-19 CE IVD lot no JN-02780-0121,UK). Laboratory tests were done and included complete blood count (CBC) with differential, assessment of CRP, D-dimer, LDH, and serum ferritin level. All patients underwent non-contrast CT chest image in the supine position at the end of inspiration using Toshiba (Zoe termer) Activion 16 Multislice CT system.

Analysis of image: The radiologist interpreted all CT images according to COVID-19 Reporting and Data System (CO-RADS) classification without knowing the clinical feature or laboratory finding. The scans were first assessed by the radiologist whether negative or positive for typical findings of COVID-19 pneumonia (bilateral, multilobar, posterior peripheral ground-glass opacities) as defined by the Radiological Society of North America (RSNA) Consensus statement followed by CO-RADS classification (Table 1) [9].

**Table 1:** CO-RADS classification [9]

	Level of suspicion for pulmonary involvement of COVID-19	Summary
CO-RADS 0	Not interpretable	Scan technically insufficient for assigning a score
CO-RADS 1	Very low	Normal or non-infectious
CO-RADS 2	Low	Typical for other infection but not COVID-19
CO-RADS 3	Equivocal/unsure	Feature compatible with COVID-19, but also other diseases
CO-RADS 4	High	Suspicious for COVID-19
CO-RADS 5	Very high	Typical for COVID-19
CO-RADS 6	Proven	RT-PCR positive for SARS-CoV-2

*CORAD* COVID-19 Reporting and Data System, *RT-PCR* real-time polymerase chain reaction

Pregnant women were excluded due to risk of CT, and also, patients with interstitial lung diseases, tuberculosis, and pulmonary malignancy were excluded to avoid interference with radiological presentation of COVID-19. All data were collected and statistically analyzed.

### Statistical analysis

The collected data were analyzed using Statistical Package for Social Science (IBM Corp. Released 2017. IBM SPSS Statistics for Windows, Version 25.0. Armonk, NY: IBM Corp.). Student’s *t* test was used to assess the statistical significance of the difference between two study group means. Mann-Whitney test (*U* test) was used to assess the statistical significance of the difference of a non-parametric variable between two study groups. Chi-square test was used to examine the relationship between two qualitative variables. Fisher’s exact test was used to examine the relationship between two qualitative variables when the expected count is less than 5 in more than 20% of cells. The receiver operating characteristic (ROC) curve was used to evaluate the sensitivity and specificity for quantitative diagnostic measures. The optimum cut off point was defined as that which maximized the area under the curve (AUC) value. AUC greater than 0.9 has high accuracy, while 0.7–0.9 indicates moderate accuracy, 0.5–0.7, low accuracy and 0.5 a chance result. Correlation analysis was used to assess the strength of association between two quantitative variables. A *p* value was considered significant if < 0.05 at confidence interval 95%.

## Results

**Fig. 1:**
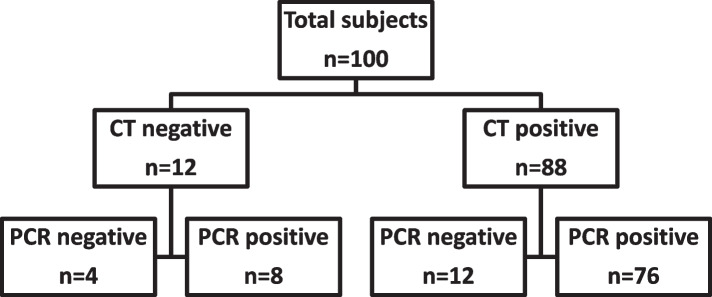
Flow chart of studied cases

One hundred patients suspected to have COVID-19 infection were involved, 88 of them had CT finding that suggest SARS-CoV infection, from those 76 patient had positive PCR. While from those who had negative CT finding, 8 patients had positive PCR test (Fig. 1).

**Fig. 2:**
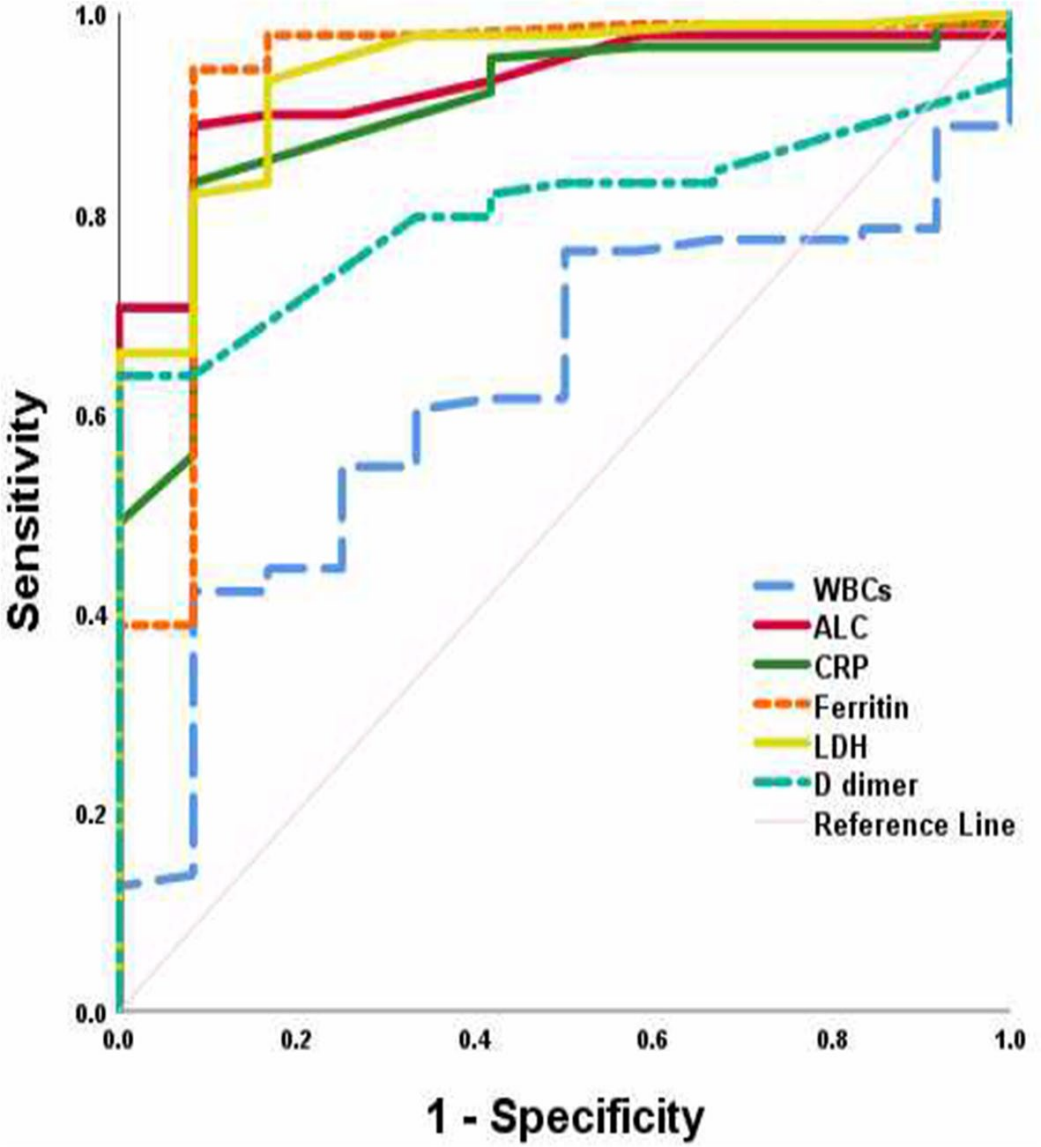
ROC curve of TLC, ALC, CRP, ferritin, LDH, and D-dimer for discrimination between negative and positive CT

The mean age of the patients was 49.6 ± 14.7 with equal sex distribution. Fever and cough were the frequent presenting symptoms. The mean lymphocyte count was 1.1 ± 0.4. The frequent CT finding included CO-RADS III and IV (Table 2). Patients with positive CT findings suggesting COVID were older, predominantly males, had lower absolute lymphocytic count (ALC) and had higher level of inflammatory biomarkers (Table 3). Patients with positive PCR were significantly associated with dyspnea, higher total leucocyte count (TLC), CRP, ferritin, LDH, and D-dimer (Table 4). Lymphopenia (ALC < 1) had sensitivity of 63.6%, specificity of 91.7% while combination of PCR and lymphopenia increased performance characteristics as shown in (Table 5). Regarding CT findings in relation to PCR, it showed sensitivity of 90.5% and specificity of 25% (Table 6). ROC curve of total TLC, ALC, CRP, ferritin, LDH, and D-dimer was conducted for discrimination between negative and positive CT. Low accuracy AUC was found for TLC, while ALC, CRP, ferritin, LDH, and D-dimer had high accuracy AUC. Best cut-off values, performance characteristics are shown in Table 7 and Fig. 2. CORADS score was found to positively correlate with age and inflammatory biomarkers while it was negatively correlated with ALC (Table 8).

**Table 2:** Patients’ features

		Total *N* = 100
Age (years)	Mean ± SD	49.6 ± 14.7
	Minimum-maximum	29–82
Males	*N*(%)	50 (50%)
Females	*N*(%)	50 (50%)
Fever	*N*(%)	71 (71%)
Cough	*N*(%)	44 (44%)
Dyspnea	*N*(%)	60 (60%)
WBCs	Mean ± SD	8.3 ± 2.6
	Minimum-maximum	1–15
ALC	Mean ± SD	1.1 ± 0.4
	Minimum-maximum	0.4–2
CRP (mg/L)	Median	9.5
	Minimum-maximum	1.5–152
FERRITIN (μg/L)	Median	425.5
	Minimum-maximum	30–2000
LDH (U/L)	Median	250
	Minimum-maximum	100–1808
D-dimer (μg/ml)	Median	0.4
	Minimum-maximum	0.1–3.2
CT	Negative	12(12%)
	Positive	88(88%)
CO-RADS	1	12(12%)
	3	29(29%)
	4	35(35%)
	5	24(24%)
RT-PCR	Negative	16(16%)
	Positive	84(84%)

*WBCs* White blood cells (4000–10000/mm^3^), *ALC* Absolute lymphocyte count (1000–4800/mm^3^), *CRP* C-reactive protein (0–5 mg/L), *Ferritin* (41–400 μg/L), *LDH* Lactate dehydrogenase (135–225 U/L), *D-dimer* (<0.5 μg/ml), *CT* computed tomography, *CORAD* COVID-19 Reporting and Data System, *RT-PCR* Real-time polymerase chain reaction

**Table 3:** Comparison of studied parameters between negative and positive CT

		Negative CT *N* = 12	Positive CT *N* = 88	*p*
Age (years)	Mean ± SD	31.8 ± 6.9	52.0 ± 13.8	< 0.001
	Minimum-maximum	26–46	29–82	
Males	*N*(%)	2 (16.7%)	48 (60.0%)	0.014
Females	*N*(%)	10 (83.3%)	40 (50.0%)	
Fever	*N*(%)	0 (0%)	71 (88.8%)	< 0.001
Cough	*N*(%)	0 (0%)	44 (55.0%)	0.001
Dyspnea	*N*(%)	0 (0%)	60 (75.0%)	< 0.001
WBCs	Mean ± SD	7.5 ± 1.7	8.5 ± 2.6	0.222
	Minimum-maximum	5–12	1–15	
ALC	Mean ± SD	1.6 ± 0.2	1.0 ± 0.3	< 0.001
	Minimum-maximum	1–2	0.4–2	
CRP (mg/L)	Median	4	10	< 0.001
	Minimum-maximum	1.7–10	1.5–152	
Ferritin (μg/L)	Median	100	450	< 0.001
	Minimum-maximum	80–595	30–2000	
LDH (U/L)	Median	125	300	< 0.001
	Minimum-maximum	100–202	100–1808	
D-dimer (μg/ml)	Median	0.24	0.45	0.020
	Minimum-maximum	0.2–0.3	0.1–3.2	
CORAD	1	12(100%)	0(0%)	< 0.001
	3	0(0%)	29(33%)	
	4	0(0%)	35(39.8%)	
	5	0(0%)	24(27.3%)	
RT-PCR	Negative	4(33.3%)	12(13.6%)	0.098
	Positive	8(66.7%)	76(86.4%)	

*WBCs* White blood cells (4000–10000/mm^3^), *ALC* Absolute lymphocyte count (1000–4800/ mm^3^), *CRP* C-reactive protein (0–5 mg/L), *Ferritin* (41–400 μg/L), *LDH* Lactate dehydrogenase (135–225 U/L), *D-dimer* (< 0.5 μg/ml), *CT* computed tomography, *CORAD* COVID-19 Reporting and Data System, *RT-PCR* Real-time polymerase chain reaction

**Table 4:** Comparison of studied parameters between negative and positive PCR

		Negative RT-PCR *N* = 16	Positive RT-PCR *N* = 84	*p*
Age (years)	Mean ± SD	43.4 ± 13.7	50.8 ± 13.9	0.065
	Minimum-maximum	9–73	16–82	
Males	*N*(%)	7 (43.8%)	43 (51.2%)	0.585
Females	*N*(%)	9 (56.3%)	41 (48.8%)	
Fever	*N*(%)	9 (56.3%)	62 (73.8%)	0.227
Cough	*N*(%)	8 (50.0%)	36 (42.9%)	0.598
Dyspnea	*N*(%)	4 (25.0%)	56 (66.7%)	0.002
WBCs	Mean ± SD	7.1 ± 1.3	8.6 ± 2.7	0.039
	Minimum-maximum	5–10	1–15	
ALC	Mean ± SD	1.1 ± 0.4	1.1 ± 0.4	0.952
	Minimum-maximum	0.5–1.8	0.4–2	
CRP (mg/L)	Median	6.5	11	0.004
	Minimum-maximum	2–55	1.5–152	
Ferritin (μg/L)	Median	200	453	0.001
	Minimum-maximum	80–650	30–2000	
LDH(U/L)	Median	190	300	0.003
	Minimum-maximum	100–350	107–1808	
D-dimer (μg/ml)	Median	0.24	0.4	0.006
	Minimum-maximum	0.1–0.9	0.1–3.2	
CT	Negative	4(25.0%)	8(9.5%)	0.098
	Positive	12(75.0%)	76(90.5%)	
CORAD	1	4(25.0%)	8(9.5%)	0.314
	3	5(31.3%)	24(28.6%)	
	4	5(31.3%)	30(35.7%)	
	5	2(12.5%)	22(26.2%)	

*WBCs* White blood cells (4000–10000/mm^3^), *ALC* Absolute lymphocyte count (1000–4800/mm^3^), *CRP* C-reactive protein (0–5 mg/L), *Ferritin* (41–400 μg/L), *LDH* Lactate dehydrogenase (135–225 U/L), *D-dimer* (< 0.5 μg/ml), *CT* Computed tomography, *CORAD* COVID-19 Reporting and Data System, *RT-PCR* Real-time polymerase chain reaction

**Table 5:** Performance characteristics of PCR and lymphopenia in relation to CT chest

	Lymphopenia	RT-PCR + lymphopenia
Sensitivity (%)	63.6	88.6
Specificity (%)	91.7	91.7
PPV (%)	98.2	88.6
NPV (%)	25.6	91.7
Accuracy (%)	67.0	89.0

*RT-PCR* Real-time polymerase chain reaction, *PPV* Positive predictive value, *NPP* Negative predictive value

**Table 6:** Performance characteristics of CT in relation to RT-PCR

	CT
Sensitivity (%)	90.5
Specificity (%)	25
PPV (%)	86.4
NPV (%)	33.3
Accuracy (%)	80

*CT* Computed tomography, *RT-PCR* Real-time polymerase chain reaction, *PPV* Positive predictive value, *NPP* Negative predictive value

**Table 7:** Validity of TLC, ALC, CRP, ferritin, LDH, and D-dimer for discrimination between negative and positive CT

	WBCs	ALC	CRP	Ferritin	LDH	D-dimer
AUC	0.625	0.927	0.900	0.931	0.940	0.801
95% CI	0.487–0.764	0.869–0.985	0.818–0.981	0.834–1	0.878–1	0.715–0.887
Cut off	7.9	1.4	5.5	135	151	0.31
Sensitivity (%)	54.5	88.6	83	94.3	93.2	63.6
Specificity (%)	75	91.7	91.7	91.7	83.3	91.7
PPV (%)	54.5	88.6	83.0	94.3	93.2	63.6
NPV (%)	75.0	91.7	91.7	91.7	83.3	91.7
Accuracy (%)	57.0	89.0	84.0	94.0	92.0	67.0

*WBCs* White blood cells (4000–10000/mm^3^), *ALC* Absolute lymphocyte count (1000–4800/mm^3^), *CRP* C-reactive protein (0–5 mg/L), *LDH* Lactate dehydrogenase (135–225 U/L), *AUC* Area under the curve, *CI* confidence interval, *PPV* Positive predictive value, *NPP* Negative predictive value

**Table 8:** Correlation of CORAD with other studied parameters

	CORAD	
	*Correlation coefficient*	*p*
Age	0.449	< 0.001
WBCs	0.196	0.051
ALC	− 0.272	0.006
CRP	0.470	< 0.001
FERRITIN	0.683	< 0.001
LDH	0.559	< 0.001
D dimer	0.533	< 0.001

*CORAD* COVID-19 Reporting and Data System, *WBCs* White blood cells (4000–10,000/mm^3^), *ALC* Absolute lymphocyte count (1000–4800/mm^3^), *CRP* C-reactive protein (0–5 mg/L), *LDH* Lactate dehydrogenase (135–225 U/L)

## Discussion

In excess of 8 million individuals were infected from COVID-19 and around 450,000 individuals passed on from COVID-19 worldwide [10]. Early determination of COVID-19 infection is vital for the prevention and control. The RT-PCR utilized as the gold standard diagnostic tool in COVID-19 illness has a few restrictions, for example, high false-negative outcomes rate, sample collection, inadequate stock of nucleic acid kits, and limited lab equipment [11].

The common discoveries in CT of COVID-19 patients were patchy, rounded segmental and sub-segmental ground glass opacities that might be worsened to consolidation [12]. In our 100 patients, the predominant symptoms were fever and dyspnea with the mean age 49.6 ± 14.7 (ranged from 29 to 82 years) with equal sex distribution. Most of patients showed lymphopenia, variable level of inflammatory markers, and positive CT finding suggesting COVID infection. Twelve percent of the studied group had negative RT-PCR result for COVID-19. Ozel and his colleagues in a similar study found that the age of their studied group was 45.9 ± 15.9 years and ranged from 18 to 91 years old, average ALC count of 1.7 ± 2.7 with minimal increases in inflammatory markers including PLT, CRP, LDH, and D-dimer. They demonstrated 60.4% of cases with negative PCR from 280 hospitalized patients diagnosed with COVID-19 pneumonia [13]. In a similar study on 200 COVID-19 patients, including 111 males and 89 females with their ages ranging from 20 to 87 years, the most common presenting symptoms were lower respiratory symptoms (66%) and fever (69.5%) [14]. No CT finding was present in 8 patients confirmed with PCR and 4 patients suspected to have COVID-19 by presence of symptoms. In a study done by Bernheim and coworkers, they found negative CT finding in 27 out of 121 patients (22%) [15].

In our study, patients with positive CT finding were significantly older, male gender, had more symptoms and significant lymphopenia with higher inflammatory markers. Also in this study, 12 patients had CT finding suggesting COVID-19 infection but with negative RT-PCR. In agreement with this finding, Zayed et al. found that dyspnea, cough, lymphopenia, CRP, and other inflammatory markers were significantly evident in patients (especially males) with severe and critically ill COVID-19 patient with evident CT finding [16]. Chen and his colleagues concluded that the more pulmonary consolidation found in CT, the greater possibility of positive initial RT-PCR [17]. The harm that occurred in the lung was strongly connected to changes in laboratory finding, even in the presence of negative RT-PCR [18]. So, diagnosis might be assumed in light of CT findings in patients with moderate to severe features of COVID-19 regardless of absence of test positivity [19].

In this study, CO-RADS score did not differ significantly between positive and negative RT-PCR. In a study done on 195 patients with COVID-19 infection (proved by positive RT-PCR) and underwent CT chest, they found that false negative result of CO-RADS reached 27.3%, and they concluded that CO-RADS system cannot be reliable to exclude the likelihood of infection [20].

On the contrary, Fujioka et al. noticed that CO-RADS maintains remarkable performance and perfect inter observer agreement by using chest CT images for predicting COVID-19 pneumonia [21].

In the current study, lymphopenia had sensitivity of 63.6% and specificity of 91.7% in diagnosis detection. Furthermore, combination of PCR and lymphocyte count increased performance characteristics to diagnose COVID-19 infection. Also, CT chest showed sensitivity of 90% and specificity of 25% in relation to PCR.

In a retrospective analysis done by Davis and Gilderman at a tertiary academic medical center in central Pennsylvania, they found that lymphopenia had a sensitivity of 13.9% and a specificity of 96% to diagnose COVID-19 infection [22]. He et al enrolled 82 hospitalized patients due to COVID-19. The sensitivity of RT-PCR and CT to identify COVID-19 were 79% and 77%, while the specificity was 100% and 96% respectively. With the initial RT-PCR plus CT strategy, the sensitivity improved to 94% and the specificity was 100% [23].

Similarly, Bai et al. demonstrated that the accuracy of three Chinese radiologists to differentiate COVID-19 from non-COVID-19 pneumonia was 83%, 80%, and 60% [24]. Another study revealed sensitivity of CT in COVID-19 is as high as 98% [25]. Similarly, a large group study of Wuhan patients announced a sensitivity of 97% for chest CT, with 308/601 patients showing representative CT manifestations before RT-PCR test was positive [26].

To discriminate between negative and positive CT finding suggesting COVID, ROC curve showed low accuracy AUC for TLC, while ALC, CRP, ferritin, LDH, and D-dimer had high accuracy AUC.

In a similar study recruited 566 individuals, it was found that CRP, LDH, ferritin and D-dimer had AUC of 0.7, 0.69, 0.73, and 0.56 respectively which increased to 0.85 when combined with 75% sensitivity and 87% specificity [27]. Also, Colak et al. concluded that as CT progressed from mild to severe, CRP, LDH, and ferritin levels gradually increased. The multinomial logistic regression analysis showed that CRP, lymphocyte, and monocyte were independent factors for COVID-19 [28].

In a systematic review done by Kermali et al., they found that CRP, LDH, D-dimer, and other inflammatory biomarkers showed significantly higher levels in patients with severe complications of COVID-19 infection. Also, lymphocytes and platelet count were significantly in lower levels in severe compared to non-severe patients. Furthermore, they presumed that biomarkers act over the span of the illness could help clinicians in distinguishing severe disease earlier and accordingly refine the prognosis [29]. Mardani and his colleagues in a study demonstrated AUC for WBCS, lymphocyte, positive CRP, and LDH of 0.075, 0.112, 0.87, and 0.835 respectively with very good accuracy in predicting cases with positive RT-PCR for COVID-19 [30].

In the current study, CO-RADS score found to have significant positive correlation with age, CRP, serum ferritin, LDH, and D-dimer while it had a negative correlation with lymphocytic count.

In a comparable study, 462 patients with positive CT scans for interstitial pneumonia due to COVID-19 were included and a significant positive correlation was found for the entire sample with lymphocytopenia, LDH, CRP, fibrinogen, and D-dimer [18].

In another study done by Patel and his colleagues on 324 patients with fever and acute respiratory symptoms suggesting COVID-19 infection, high-resolution CT (HRCT) was done in addition to RT-PCR and laboratory tests. They observed that ferritin, CRP, and LDH were significantly higher in patients with COVID-19 whose CT showed distinct multifocal pneumonia and extensive ground glass opacity (GGO) while platelet and ALC were significantly low [31]. Also, Abdelmoty et al found significant validity of lymphopenia, increased D-dimer, LDH, CRP, and serum ferritin to predict severity of COVID-19 infection [32]. On the contrary, Sadek and his colleagues found no correlation between CO-RADS score and different CBC parameters; neither lymphopenia nor high neutrophil/lymphocyte ratio predicts the CO-RADS score [33].

### Study limitations

This study was performed in a single center. Likewise, the numbers of cases in the CO-RADS categories were not equal (with no significant difference) and generally small. Furthermore, 16% of participants had negative RT-PCR test results. Still, the false negative is usual for PCR in COVID-19.

## Conclusion

CT findings suggesting COVID-19 infection were more prominent in older patients and associated with higher inflammatory biomarkers and lower ALC which correlate significantly with CO-RADS score. Patients with positive PCR had increased inflammatory marker. Combination of PCR with either lymphopenia or CT finding had more sensitivity, specificity, and accuracy in diagnosis.

## Data Availability

Not applicable.

## Abbreviations

SARS-Co V-2: Severe acute respiratory syndrome coronavirus 2
PCR: Polymerase chain reaction
COVID-19: Coronavirus Disease 2019
IL-6: Interleukin-6
GLU: Glucose
TT: Thrombin time
FIB: Fibrinogen
CRP: C-reactive protein
LDH: Lactate dehydrogenase
ICU: Intensive care unit
NEU: Neutrophil
CT: Computed tomography
VTM: Viral transport medium
RNA: Ribonucleic acid
RT-PCR: Real-time polymerase chain reaction
CO-RADS: COVID-19 Reporting and Data System
ROC: Receiver operating characteristic
AUC: Area under the curve
TLC: Total leucocyte count
ALC: Absolute lymphocyte count
PCT: Procalcitonin
HRCT: High-resolution computed tomography
GGO: Ground glass opacity
